# Aplastic anemia induced disc edema and visual loss in pregnancy: a case report

**DOI:** 10.1186/1757-1626-1-322

**Published:** 2008-11-18

**Authors:** Shailesh K Gupta, Vikram S Brar, Ravi Keshavamurthy, Kakarla V Chalam

**Affiliations:** 1 University of Florida-College of Medicine, Department of Ophthalmology, Jacksonville, Florida, USA

## Abstract

**Introduction:**

A case of aplastic anemia diagnosed during pregnancy, which developed bilateral disc edema and acute pre-retinal hemorrhage leading to vision loss.

**Case Presentation:**

A 20 year old primagravid female developed acute vision loss in her right eye, during hospitalization for treatment of aplastic anemia diagnosed during her pregnancy. Her best-corrected visual acuity (BCVA) was hand motions and fundus evaluation revealed a large pre-macular hemorrhage in the right eye (OD) and bilateral disc edema. Neuro-imaging studies did not reveal any signs of intracranial mass lesion or edema.

**Conclusion:**

There was resolution of the disc edema with improvement in the pre-macular hemorrhage resulting in 20/50 vision in the right eye, following supportive transfusions. Ophthalmic manifestations developing in a pregnant patient with aplastic anemia can be successfully managed with supportive care including red blood cell and platelet transfusions.

## Introduction

Aplastic anemia, a serious hematological disorder characterized by pancytopenia and hypoplastic bone marrow is often exacerbated during pregnancy [1-3]. Hormonal imbalance between hematopoietic placental lactogen and erythropoietin and marrow suppressive estrogen result in this association [3]. We report an unusual case of bilateral disc edema and visual loss due to pre-retinal hemorrhage in a patient with aplastic anemia, diagnosed during pregnancy.

## Case presentation

A 20 year-old primagravid Caucasian female at 20 weeks of gestation presented to the emergency department with complaints of weakness, dizziness, headaches and palpitations. Complete blood count (CBC) analysis revealed white blood cell count (WBC) of 1.9 × 10^9 ^cells/litre, hemoglobin of 4.9 gm/dl, and platelet count of 5 × 10^9 ^cells/litre. Further investigation showed normal liver function tests and prothrombin time and an INR of 13.3s and 0.9 respectively. Bone marrow biopsy confirmed the etiology of the pancytopenia as aplastic anemia and subsequent red blood cell and platelet transfusions stabilized the patient's hematologic status.

**Figure 1:**
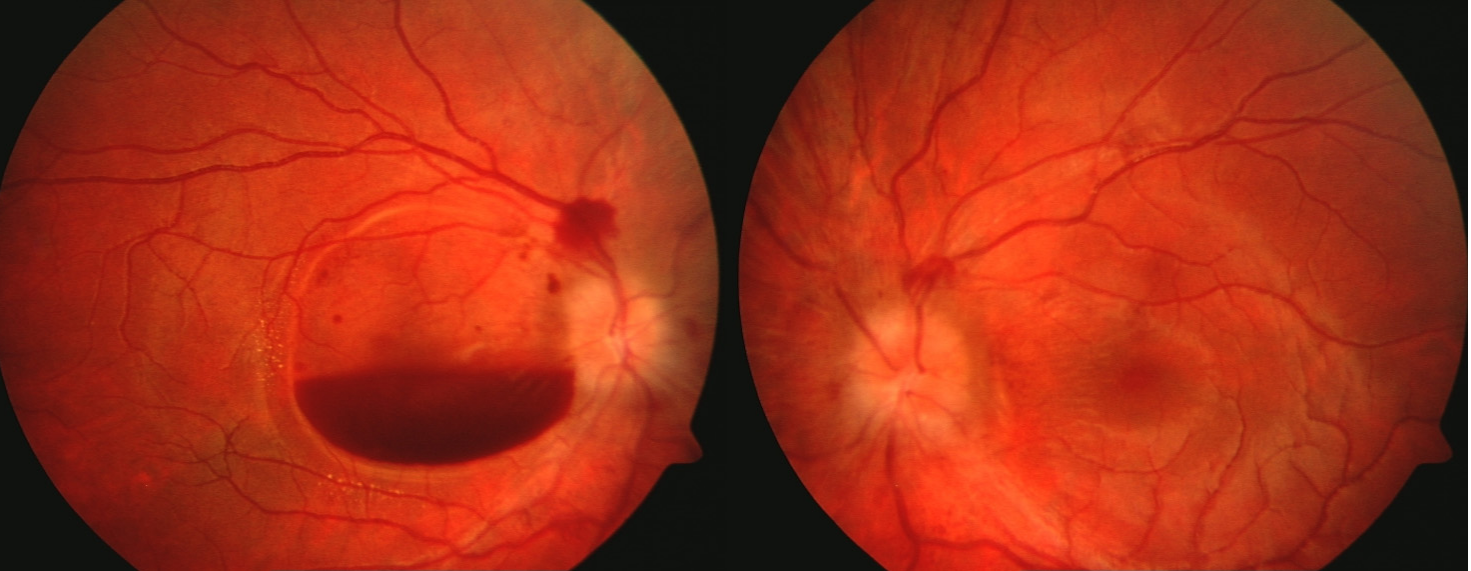
**Color fundus photograph depicting layered pre-retinal hemorrhage in the right eye and disc edema.** Peri-papillary nerve fiber layer hemorrhages are also present.

**Figure 2:**
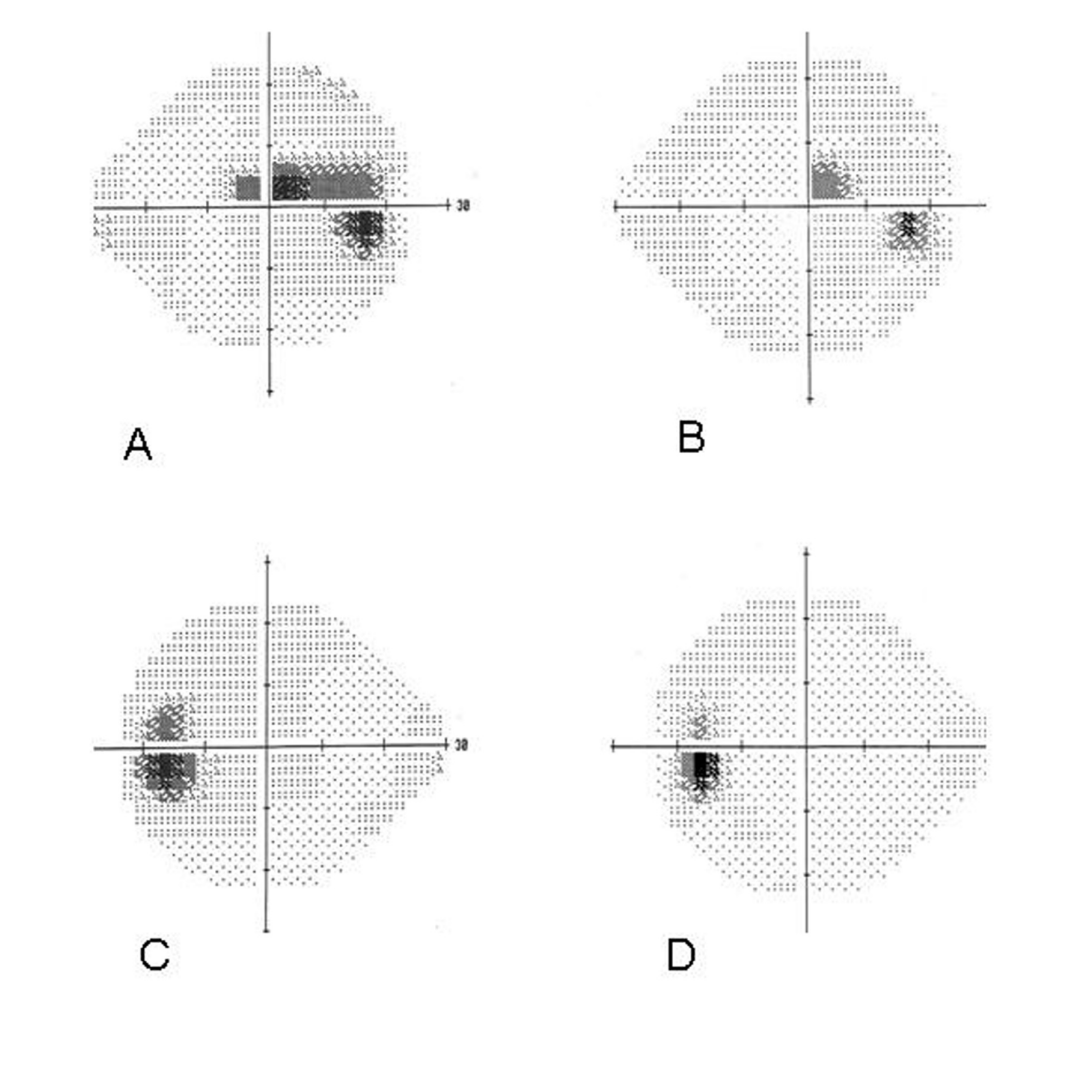
**(A) 24-2 Humphrey visual field (HVF) revealing enlarged blind spot with a superior paracentral scotoma in the right eye (mean deviation -4.02 db).** (B) Follow up 24-2 HVF demonstrating normal blind spot and improved superior paracentral scotoma in the right eye (mean deviation -2.63 db). (C) 24-2 HVF revealing enlarged blind spot in the left eye (mean deviation -2.79 db) (D) Follow up 24-2 HVF demonstrating normal blind spot (mean deviation (-0.74 db).

During her initial hospitalization, she reported decreased vision in her right eye which she described as a red spot when looking at the light. On ophthalmic consultation, her visual acuity was hand motions (HM) in the right eye and 20/20 in the left eye, with normal intraocular (IOP) in both eyes. Anterior segment examination was unremarkable and pupillary reactions were normal. Dilated fundoscopic examination done at that time had revealed bilateral optic disc swelling and a layered pre-retinal hemorrhage involving the macula of her right eye (Figure 1). Humphrey visual field examination (HVF) revealed enlarged blind spots in both the eyes with a ceco-central defect (mean deviation -4.02 db) in the right superotemporal quadrant, correlating with the retinal hemorrhage present clinically (Figures 2A,C). Neuro-imaging, including both CT and MRI, did not reveal a mass lesion and lumbar puncture resulted in a dry tap in spite of repeated attempts.

**Figure 3:**
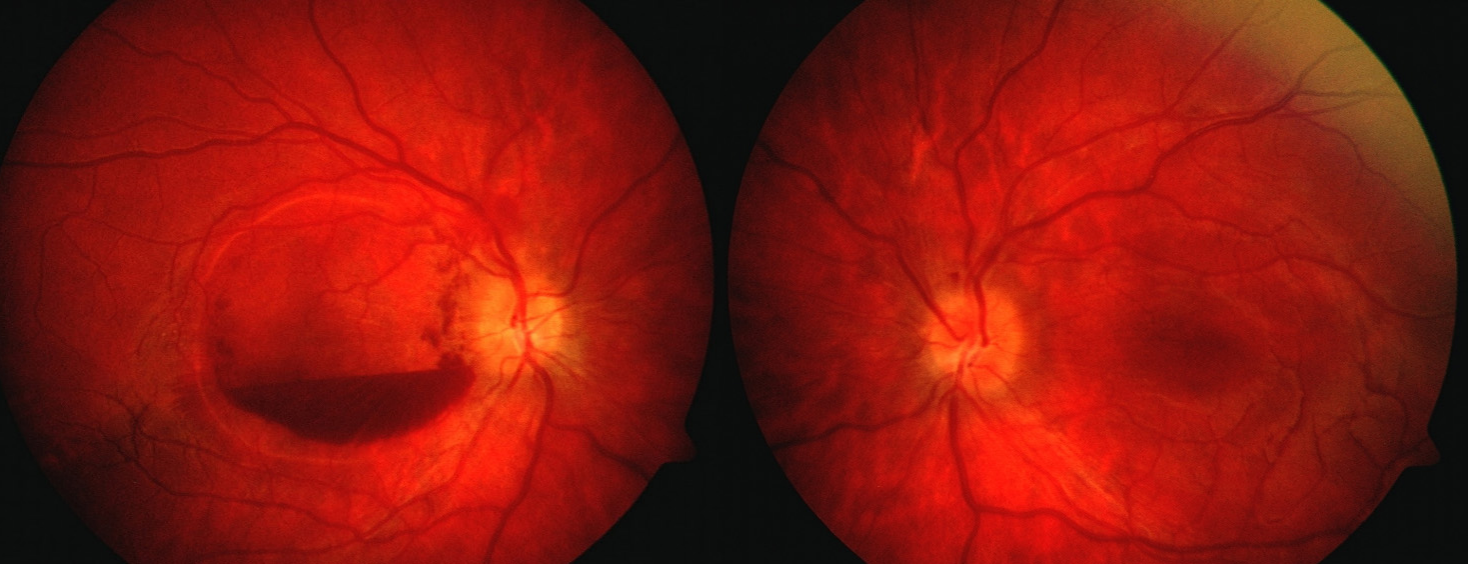
Color fundus photograph showing resolved disc edema in both eyes and central clearing of the pre-retinal hemorrhage in the right eye.

The visual acuity in the right eye and the disc edema gradually improved. During this course, the patient's hematologic status and fetus were closely monitored, with administration of supportive transfusions as needed. The patient went on to deliver a 1664 gram infant at 32 weeks gestation. On post-partum day 2, her visual acuity was 20/50 and 20/20 in the right and left eyes, respectively. Dilated fundoscopic examination revealed resolving pre-retinal hemorrhage, clearing from the visual axis, with resolution of the disc edema bilaterally (Figure 3). Repeat Humphrey visual field testing exhibited normalization of the blind spot in both the eyes, with improvement in the paracentral scotoma of the right eye (mean deviation -2.63 db) correlating with the remaining pre-retinal hemorrhage (Figures 2B, D).

## Discussion

The first reported case of aplastic anemia was in a pregnant individual in 1888 [4]. Other conditions associated with aplastic anemia include idiosyncratic drug reactions (chloramphenicol), chemical exposure, eosinophilic fasciitis, and seronegative hepatitis [5]. 78% of cases of aplastic anemia exhibit ophthalmic manifestations. Typical ophthalmic manifestations include eyelid hematoma, subconjunctival hemorrhage, cotton wool spots, retinal nerve fiber layer hemorrhage, Roth's spots, pre-retinal hemorrhage, vitreous hemorrhage, and disc edema. Of these, retinal hemorrhage and cotton wool spots are the most common, 67% and 38% respectively [6].

Vision loss in our case is secondary to pre-retinal hemorrhage overlying the fovea in the right eye. Visual impairment secondary to pre-retinal hemorrhages can be a presenting symptom in previously undiagnosed cases of aplastic anemia [6]. The presence of hemorrhages in the setting of anemia and thrombocytopenia has been described as part of the constellation of retinal findings in patients with anemic-thrombocytopenic retinopathy. Carraro et al reported an increased prevalence of retinopathy in patients with hemoglobin levels <8 mg/dL and platelet counts <50 × 10^9^/L, which is the case for many of the patients affected by aplastic anemia. 2% of patients with retinal hemorrhages exhibited pre-retinal lesions and 1 of 65 patients with ocular findings exhibited bilateral disc edema [7].

Optic disc edema has been reported to occur in 6% of cases of aplastic anemia, with the etiology most likely being related to elevated intracranial pressure [6]. Two separate case reports describe the occurrence of idiopathic intracranial hypertension in 3 adolescent patients with aplastic anemia, which responded to management with acetazolamide and correction of the anemia [8,9]. A recent retrospective case series further highlighted this relationship by describing 7 cases of improvement of papilledema associated with anemia by correction of anemia alone. In another case from the same series, the patient's papilledema only responded to the correction of the anemia despite prior interventions to lower the intracranial pressure [10]. In our patient, we could not establish raised intracranial pressure as the cause for optic disc edema as the lumbar puncture resulted in a dry tap. None the less, our patient's disc edema and enlarged blind spot on HVF testing gradually improved as the hemoglobin levels stabilized.

## Conclusion

In summary, this report illustrates a case of visual loss associated with pregnancy related aplastic anemia which was successfully managed with supportive care including red blood cell and platelet transfusions.

## Abbreviations

CBC: Complete blood count; BCVA: Best-corrected visual acuity; WBC: White blood cell count; IOP: Intraocular pressure; CT: Computed Tomography; MRI: Magnetic Resonance Imaging.

## Consent

Written informed consent was obtained from the patient for publication of this case report and any accompanying images. A copy of the written consent is available for review by the Editor-in-Chief of this journal.

## Competing interests

The authors declare that they have no competing interests.

## Authors' contributions

KVC and SG identified the case and directly participated in management. They also revised the manuscript and verified its intellectual content. VSB and RK worked in collaboration to collect data, acquire clinical photographs, and draft, revise, and reference the manuscript.
